# Lycopene inhibits IL‐1β‐induced inflammation in mouse chondrocytes and mediates murine osteoarthritis

**DOI:** 10.1111/jcmm.16443

**Published:** 2021-03-10

**Authors:** Jingdi Zhan, Zijian Yan, Xiaojiang Kong, Junling Liu, Zeng Lin, Weihui Qi, Yifan Wu, Jian Lin, Xiaoyun Pan, Xinghe Xue

**Affiliations:** ^1^ Department of Orthopaedics The Second Affiliated Hospital and Yuying Children’s Hospital of Wenzhou Medical University Wenzhou China; ^2^ Zhejiang Provincial Key Laboratory of Orthpaedics Wenzhou China; ^3^ The Second School of Medicine Wenzhou Medical University Wenzhou China

**Keywords:** lycopene, NF‐κB, Nrf2/HO‐1, osteoarthritis, STAT3

## Abstract

Osteoarthritis (OA) is a common chronic degenerative condition in the elderly, in which inflammation plays a key role in disease pathology. Lycopene (Lye), a member of the carotenoid family, has been reported to have anti‐inflammatory effects. The purpose of this study was to investigate the effect of Lye on the inflammation of chondrocytes and the mouse OA model. Chondrocytes were treated with interleukin (IL)‐1β, and the mouse OA model was induced by the surgical destabilization of the medial meniscus (DMM). The results showed that Lye could inhibit the expression of inflammatory factors and alleviate the degradation of extracellular matrix (ECM). Additionally, Lye could activate the Nrf2/HO‐1 pathway and reverse the activations of NF‐κB and STAT3 signal pathway induced by IL‐1β, suggesting that its anti‐inflammatory effect may be mediated via these pathways. The animal experiments showed that Lye could decrease the Osteoarthritis Research Society International (OARSI) scores of the knee, indicating that it could inhibit the occurrence and development of OA in mouse. Overall, our results indicated that Lye might be used as a novel drug for OA treatment.

## 1 INTRODUCTION

Osteoarthritis (OA) is a chronic progressive disease that occurs most frequently in the elderly.^1^, ^2^ The disease development begins from initial pain to the disorder of joint movement, that can result in joint replacement surgery, owing to its serious impact on the quality of life.^3^, ^4^ On the other hand, although OA is very common, there is still no effective treatment.^5^ Current studies suggest that inflammation plays a momentous role in the occurrence and development of OA.^6^, ^7^, ^8^ Some inflammation‐related factors, such as inducible nitric oxide synthase (iNOS), tumour necrosis factor‐α (TNF‐α), cyclooxygenase‐2 (COX‐2) and interleukin‐6 (IL‐6) have been confirmed to be overexpressed in OA.^9^, ^10^ These inflammatory factors can facilitate the degradation of extracellular matrix (ECM), which play an important role in the development of OA.^11^ Additionally, this could also accelerate the degeneration and wearing of cartilage causing hyperosteogeny, which would aggravate the clinical symptoms.^3^ It has also been reported that a disintegrin and metalloproteinase with thrombospondin‐5 (ADAMTS‐5) and matrix metalloproteinase‐13 (MMP‐13) are highly expressed in OA. These cytokines are the main indicators of catabolism in articular chondrocytes.^6^, ^7^, ^10^

**FIGURE 1:**
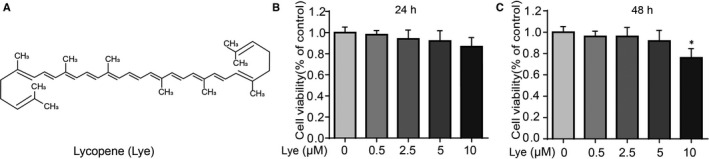
Effect of Lye on chondrocytes viability. A, Chemical construction of Lye. B, C, The cytotoxicity of Lye on chondrocytes was analysed at various concentrations for 24 and 48 h by CCK‐8 assay. The values presented means ± SD five independent experiments. **P < 0.01, *P < 0.05 vs. control group, n = 5

Lycopene (Lye) is a member of the carotenoids family.^12^, ^13^ It is mainly found in fruits and vegetables such as tomatoes (especially the red variety), watermelon and red pomelo.^14^, ^15^ The chemical structure of Lye is an acyclic carotenoid with 11 linear conjugated double bonds (Figure 1A).^13^ In recent years, interest in Lye has increased rapidly, because the studies have shown that carotenoids have certain beneficial effects on human health and diseases, such as cardiovascular protection,^16^ inhibiting the proliferation of malignant tumours such as prostate cancer and breast cancer.^17^, ^18^, ^19^ It had also been reported that Lye could not only play an anti‐inflammatory role in the animal models, but also can prevent the spermatogenic disorders and nephrotoxicity.^20^, ^21^, ^22^, ^23^, ^24^ The mechanism involved may include the Nrf2 pathway that gets regulated to exert an anti‐inflammatory and antioxidant effect.^25^ Additionally, some literatures suggest that Lye could down‐regulate the TNF‐α and IL‐6, inhibit the phosphorylation of NF‐κB and enhance the expression of Nrf2.^26^, ^27^ However, there are only few studies on Lye in OA. Therefore, the purpose of this study is to investigate the anti‐inflammatory effect of Lye in vitro, on chondrocytes and in vivo, on mouse DMM model, and the related signalling pathway mechanism.

## 2 MATERIALS AND METHODS

### 2.1 Reagents

Lycopene (Lye, purity ≥98%) was obtained from Solarbio. Antibodies against Collagen II, GAPDH, HO‐1, MMP‐13, Lamin B, were purchased from Abcam. Antibodies against COX‐2, iNOS, p65, IκBα, Nrf2, STAT3 and p‐STAT3 were supplied by Cell Signaling Technology. Alexa Fluor^®^488‐labelled, Alexa Fluor^®^594‐labelled and Goat Anti‐Rabbit IgG was purchased from Jackson ImmunoResearch.

### 2.2 Isolation and culturing of chondrocytes

The knee cartilage of C57BL/6 mouse was isolated and then digested using 5‐10 mL of 2 mg/mL type‐II collagenase (Sigma‐Aldrich) at 37°C for 4 hours. After centrifugation, the supernatant was inoculated in a petri‐dish with DMEM/F12 (Gibco) contained 10% foetal bovine serum (FBS) and 1% penicillin/streptomycin (P/S) antibiotics (Gibco). The chondrocytes were then cultured at 37°C with 5% CO_2_. The cells of passage# 2 were used for subsequent experiments.

### 2.3 Animal model

Forty‐five 10‐week‐old C57BL/6 male mice were purchased from Animal Center of Chinese Academy of Sciences Shanghai, China. The experimental protocols for animal use and care were based on the Guidelines for the Care and Use of Laboratory Animals of the National Institutes of Health and were approved by Wenzhou Medical University Animal Care and Use Committee (ethic code: 2018‐078). Forty‐five mice were randomly divided into three groups, each with 15 mice. Group 1 was the ‘control group’ that underwent an arthrotomy without the surgical destabilization of the medial meniscus (DMM); Group 2 was the ‘OA group’, which underwent DMM as per the previous literature^28^; Group 3 was ‘OA group treated with Lye’, which underwent DMM and was intragastric administration of Lye. In order to compare the anti‐inflammatory effect of Lye with that of classic anti‐inflammatory agents, ibuprofen was set as the positive control group.

### 2.4 Experimental design

IL‐1β (10 ng/mL) was used alone or in combination with Lye at different concentrations (0.5, 2.5, 5.0 μM) to observe the in vitro anti‐inflammatory effect on chondrocytes. Additionally, Mg132 is a commonly used proteasome inhibitor that could inhibit the activation of NF‐κB pathway. In vitro experiment of this study, MG132 (5μM) was selected as a positive control to compare the inhibitory effect of Lye on NF‐κB pathway, so as to further investigate the influence of Lye on OA.

For in vivo studies, DMM was performed on mice to cause knee instability, followed by intragastric treatment of Lye (5 mg/kg/d) in the treatment group, once a day for 8 weeks. Meanwhile, the OA group and control group were given intragastric treatment of an equivalent volume of physiological saline. And the positive control group was given intragastric treatment of ibuprofen (5 mg/kg/d). All animals were sacrificed 8 weeks after surgery, and tissue from the knee cartilage was collected for histological analysis.

### 2.5 Cell viability

The cell counting kit‐8 (CCK‐8; Dojindo Co) was used to analyse the cytotoxic effect of Lye on the chondrocytes. Chondrocytes were transferred to a 96‐well plate (5 × 10^3^/well) and treated with different concentration of Lye (0.5, 2.5, 5.0, 10 μM) for 24 and 48 hours. Then, 10 μL of the CCK‐8 solution was added to each well and the 96‐well plate was incubated at 37°C for 2 hours. Finally, the absorbance was determined at 450 nm.

### 2.6 The measurement of nitric oxide (NO) and ELISA

The measurement of NO was performed by measuring its metabolite (nitrite) using Griess reagent.^29^ The cytokine concentrations were analysed by ELISA kits (R&D Systems).

### 2.7 Immunofluorescence

Chondrocytes were planted on glass slides in a 6‐well plate (5 × 10^4^/well) and cultured for 24 hours. Cells were then incubated with or without Lye (5.0 μM) for the next 24 hours. Next, the cells were co‐cultured with IL‐1β (10 ng/mL) for 24 hours. After that, the treated chondrocytes were fixed with 4% paraformaldehyde for 15 minutes and then treated with 0.1% Triton X‐100 at room temperature for 10 minutes. The 5% goat serum was used to block the cells that were kept in a wet box at 37°C for 1 hour. These were then incubated with primary antibodies as follows: collagen II (1:300), p65 (1:200), MMP‐13 (1:200) and Nrf2 (1:100) overnight at 4°C in a wet box. After 12 hours, the cells were treated with secondary antibodies—Alexa Fluor^®^488 (1:500) conjugated or Alexa Fluor^®^594 (1:500) conjugated at room temperature for 45 minutes in a darkroom. Finally, the cell nucleus was stained using DAPI. After each step, the cells were washed using phosphate buffered saline (PBS). For histological analysis, immunofluorescence was also performed. Dewaxed histological sections were placed in citrate antigen retrieval solution (0.01 M, pH 6.0; Wanlei, China). This solution was maintained at a sub‐boiling temperature by microwaving it for 10 minutes and letting it cool on bench top for 30 minutes. Subsequent protocol followed was similar to the steps followed for immunofluorescence staining of the cells. After completion of staining, five visual fields were randomly chosen on each glass slide, and each of these fields were observed and recorded by fluorescence microscope (Olympus Inc). The intensity of fluorescence was measured by ImageJ software.

### 2.8 Western blotting

A total of 2 × 10^6^‐2 × 10^7^ chondrocytes were cultured, washed thrice with pre‐cooled PBS, then dissociated with 0.05% trypsin and collected by centrifugation. The nuclear and cytoplasmic extraction was performed with a Nuclear and Cytoplasmic Protein Extraction Kit (Wanlei, China). Briefly, the cells precipitation was lysed in ice‐cold cytoplasmic extraction reagent and incubated on ice for 15‐20 minutes. The next step was to centrifuge at 13 800 g for 5 minutes at 4°C, the supernatant was the cytoplasmic protein. Next, 50 μL of nuclear extraction reagent was added to the rest of the precipitate and incubated on ice for 30 minutes. Nuclear proteins were harvested in the supernatant after centrifugation at 13 800 g for 10 minutes at 4°C. The total protein of chondrocytes from treated and control groups were extracted and the BCA protein assay kit (Beyotime) was used to analyse its concentration. Total protein of 50 μg was separated into multiple target protein using sodium dodecyl sulphate‐polyacrylamide gel electrophoresis (SDS PAGE) and then transferred into a polyvinylidene difluoride membrane (Bio‐Rad). The 5% skimmed milk was used to block membrane for 2 hours at room temperature, and then, it was immersed in the primary antibodies diluted solution overnight at 4°C. These included the antibodies against STAT3 (1:1000), p‐STAT3 (1:1000), GADPH (1:3000), Nrf2 (1:500), HO‐1 (1:1000), iNOS (1:800), COX‐2 (1:800), IκBα (1:1000), Lamin B (1:1000), collagen II (1:1000), MMP‐13 (1:800) and p65 (1:1000).

### 2.9 Real‐time PCR

The total RNA of chondrocytes treated with IL‐1β (10 ng/mL) and Lye (at different concentrations) was obtained using TRIzol reagent (Invitrogen). The cDNA was synthesized by reverse transcription of 1000 ng of total RNA (MBI Fermentas). Parameters for RT‐PCR were 95°C for 10 minutes, followed by 40 cycles of 95°C for 15 seconds and 60°C for 1 minute. The CFX96 Real‐Time PCR System (Bio‐Rad, USA) was used for this reaction. The cycle threshold (Ct) values was obtained and normalized to the level of GAPDH.

The levels of relative mRNA of each target gene was calculated. The primers of iNOS, COX‐2, TNF‐α and IL‐6 were as follows: iNOS (F) 5′‐ GACGAGACGGATAGGCAGAG‐3′, (R) 5′‐ CACATGCAAGGAAGGGAACT‐3’; COX‐2 (F) 5′‐ TCCTCACATCCCTGAGAACC‐3′, (R) 5′‐ GTCGCACACTCTGTTGTGCT‐3′; TNF‐α (F) 5′‐ACGGCATGGATCTCAAAGAC‐3′, (R) 5′‐ GTGGGTGAGGAGCACGTAGT‐ 3′; IL‐6, (F) 5′‐CCGGAGAGGAGACTTCACAG‐3′, (R) 5′‐ TCCACGATTTCCCAGAGAAC‐3′, and which were designed with reference to NCBI Primer‐Blast Tool.

### 2.10 siRNA transfection

The Nrf2‐small interfering RNA (siRNA) were procured from Invitrogen. The sequences of Nrf2‐siRNA were as follows: sense, 5’‐UUGGGAUUCACGCAUAGGAGCACUG‐3’; antisense, 5’‐CAGUGCUCCUAUGCGUGAAUCCCAA‐3’. The Lipofectamine™ RNAiMAX Reagent was used to transfect the Nrf2 and negative control (NC) siRNA to chondrocytes as per manufacturer's instruction.

### 2.11 Histopathologic analysis

The safranin O‐fast green (S‐O) was used for staining the sections of knee joint. The stained sections were graded under the microscope by professional histology researchers using the Osteoarthritis Research Society International (OARSI) scoring system.^30^ The total OARSI score is the sum of the tibial plateau and the femoral condyle. Scores in each quadrant range from 0 to 6. A score of 0 represents normal cartilage, 0.5 = loss of proteoglycan with an intact surface, 1 = superficial fibrillation without loss of cartilage, 2 = vertical clefts and loss of surface lamina (any % or joint surface area), 3 = vertical clefts/erosion to the calcified layer lesion for 1%‐25% of the quadrant width, 4 = lesion reaches the calcified cartilage for 25%‐50% of the quadrant width, 5 = lesion reaches the calcified cartilage for 50%‐75% of the quadrant width, and 6 = lesion reaches the calcified cartilage for >75% of the quadrant width.^30^ Fifteen mice from each group were used for histomorphometric scoring.

### 2.12 Statistical analysis

The experiments were repeated at least 5 times. The analysis of the data was performed with SPSS 18.0 software. One‐way analysis of variance (ANOVA) was utilized for the data analysis, followed by the Tukey's test for comparison between the groups. The results were depicted as mean ± SD The *P*‐values below 0.05, for the differences between groups, were considered as statistically significant.

## 3 RESULTS

### 3.1 Effect of Lye on the chondrocyte viability

The chemical formula of Lye is C_40_H_56_, and the chemical structure is as presented in Figure 1A. In order to analyse the cytotoxicity of Lye on chondrocytes, chondrocytes were treated with different concentrations of Lye (0, 0.5, 2.5, 5.0, 10 μM) for 24 and 48 hours, and then CCK‐8 assay was used to observe the cell viability. As shown in Figure 1B,C, compared with the control group, Lye significantly decreased the viability of chondrocytes at the concentration of 10 μM after 48 hours (*P* < 0.05). When the concentration of Lye was ≤5 μM, whether at 24 or 48 hours, it showed no obvious cytotoxicity on chondrocytes. Based on these data, the subsequent in vitro experiments used Lye concentrations of 0.5, 2.5 and 5.0 μM for 24 hours.

### 3.2 Lye attenuated the expression of iNOS, COX‐2, TNF‐α, IL‐6, PGE2 and NO in chondrocytes under the stimulation of IL‐1β

**FIGURE 2:**
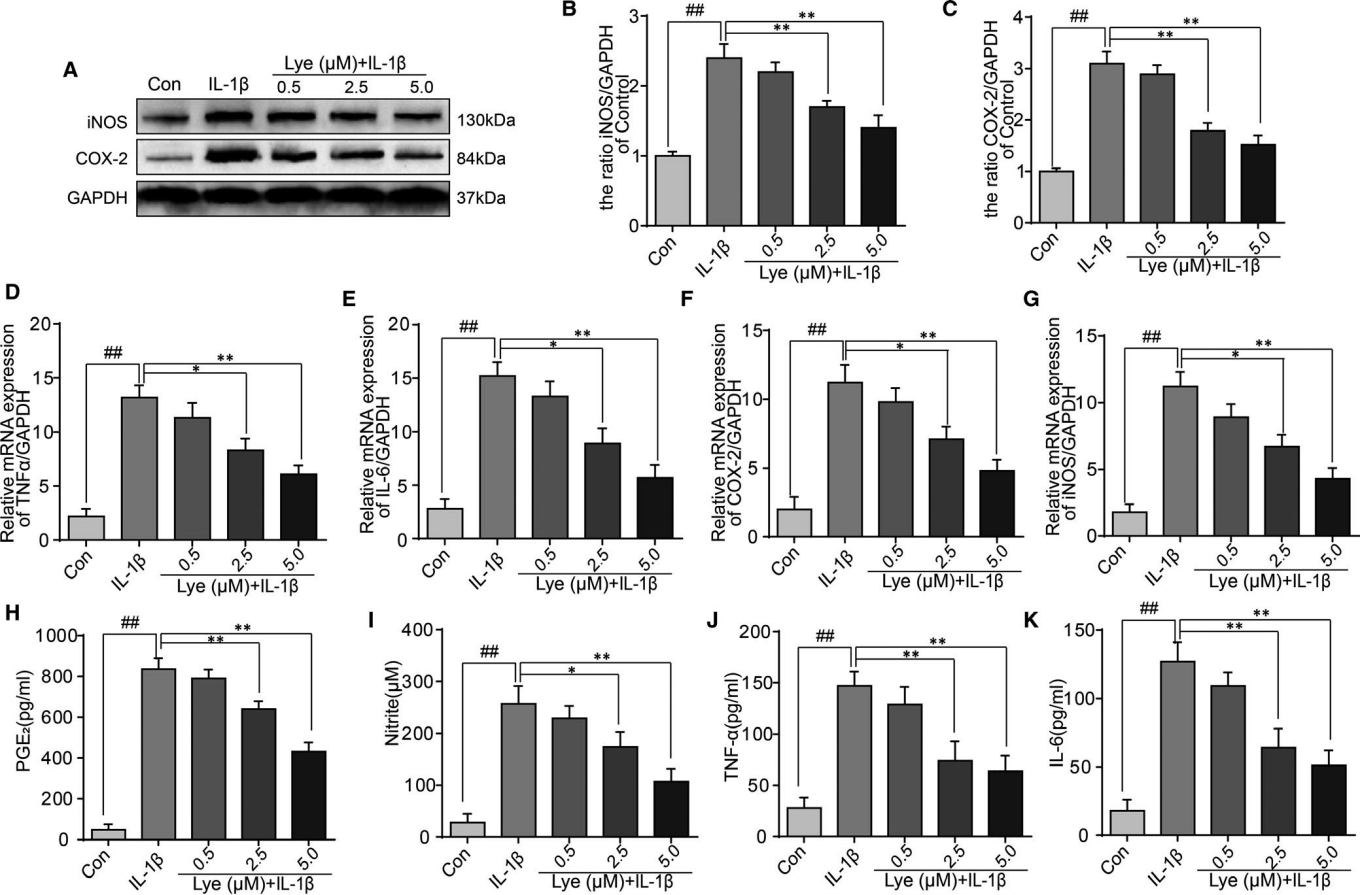
Lye attenuates the inflammation in chondrocytes under the stimulation of IL‐1β. A‐C, The expressions of iNOS and COX‐2 at protein level were detected by Western blot with different concentrations of Lye. D‐G, The expression of mRNA of TNF‐α, IL‐6, COX‐2 and iNOS were measured via real‐time PCR. H‐K, Effects of Lye on production of IL‐1β‐induced PGE2, NO, TNF‐α and IL‐6 were detected by ELISA. All data represent Mean values ± SD ##*P* < 0.01, compared with control group; *P < 0.05, **P < 0.01, compared with IL‐1β treatment group, n = 5

The expressions of iNOS, COX‐2, TNF‐α, IL‐6, PGE_2_ and NO were analysed using RT‐PCR, Western blot, ELISA or Griess reagent to observe the effect of Lye on chondrocytes when cells were stimulated by IL‐1β. As shown in Figure 2A‐C, stimulation by IL‐1β significantly up‐regulated the expressions of iNOS and COX‐2 at protein level, while the expressions was reversed by Lye in a concentration‐dependent manner. We can see from the Figure 2D‐G, at mRNA level, the expressions of TNF‐α, IL‐6, iNOS and COX‐2 were similar to that of its protein level. This also suggested that IL‐1β could increase their expression, while Lye could inhibit this process. The results of ELISA and Griess reagent (Figure 2H‐K) further demonstrated that Lye attenuated the IL‐1β‐induced elevation of nitrite, TNF‐α, IL‐6 and PGE_2_. From the results in Figure S1a,c,d,f‐l, the expression of inflammatory factors (iNOS, COX‐2 etc) in both MG132 group and Lye group was significantly different from that in IL‐1β group, and there was no significant difference between MG132 group and Lye group. Combined with the above data, when the concentration of Lye was 2.5 or 5.0 μM, it could prominently inhibit the increase in inflammatory factors caused by IL‐1β at the gene and protein levels.

### 3.3 Lye attenuated the IL‐1β‐induced degradation of ECM in mouse chondrocytes

**FIGURE 3:**
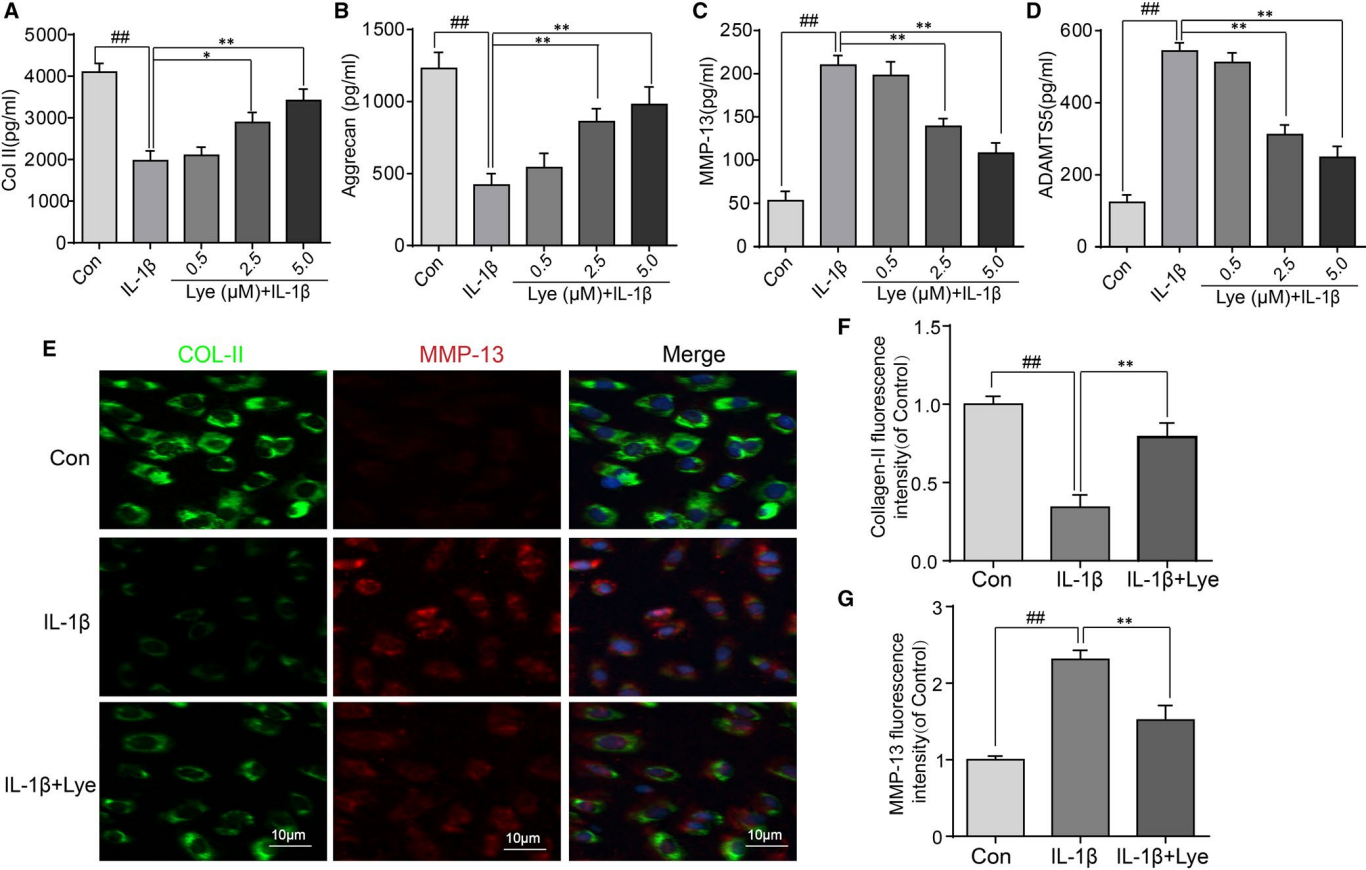
Attenuation caused by Lycopene to IL‐1β‐induced degradation of ECM in mouse chondrocytes. A‐D, The trends of Collagen II, aggrecan MMP‐13 and ADAMTS‐5 expression with various treatments were detected by ELISA. E, The immunofluorescence staining of Collagen II and MMP‐13 staining in chondrocytes (scale bar: 10 μm). F, G, The Collagen II and MMP‐13 fluorescence intensity were analysed by ImageJ software. Significant differences among different groups were indicated as ##*P* < 0.01, compared with control group; *P < 0.05, **P < 0.01, compared with IL‐1β alone treatment group, n = 5

The expressions of aggrecan, ADAMTS5, collagen II and MMP‐13 detected by ELISA were utilized to assess the effect of Lye on IL‐1β‐induced degradation of ECM. Figure 3A,B and Figure S1m,n show that the expressions of collagen II and aggrecan were evidently reduced by stimulation of IL‐1β; however, it could be reversed when Lye and MG132 were added. The trends of MMP‐13 and ADAMTS5, as Figure 3C,D and Figure S1o,p show, were opposite to that of the collagen II and aggrecan. Additionally, the changes of these factors in Lye group were not significantly different from those in Mg132 group. Furthermore, the results of immunofluorescence assay of the combination of collagen II and MMP‐13 were not contradicting to the trend seen in ELISA (Figure 3E‐G).

### 3.4 Lye repressed activation of NF‐κB and STAT3 pathway which induced by IL‐1β in chondrocytes

**FIGURE 4:**
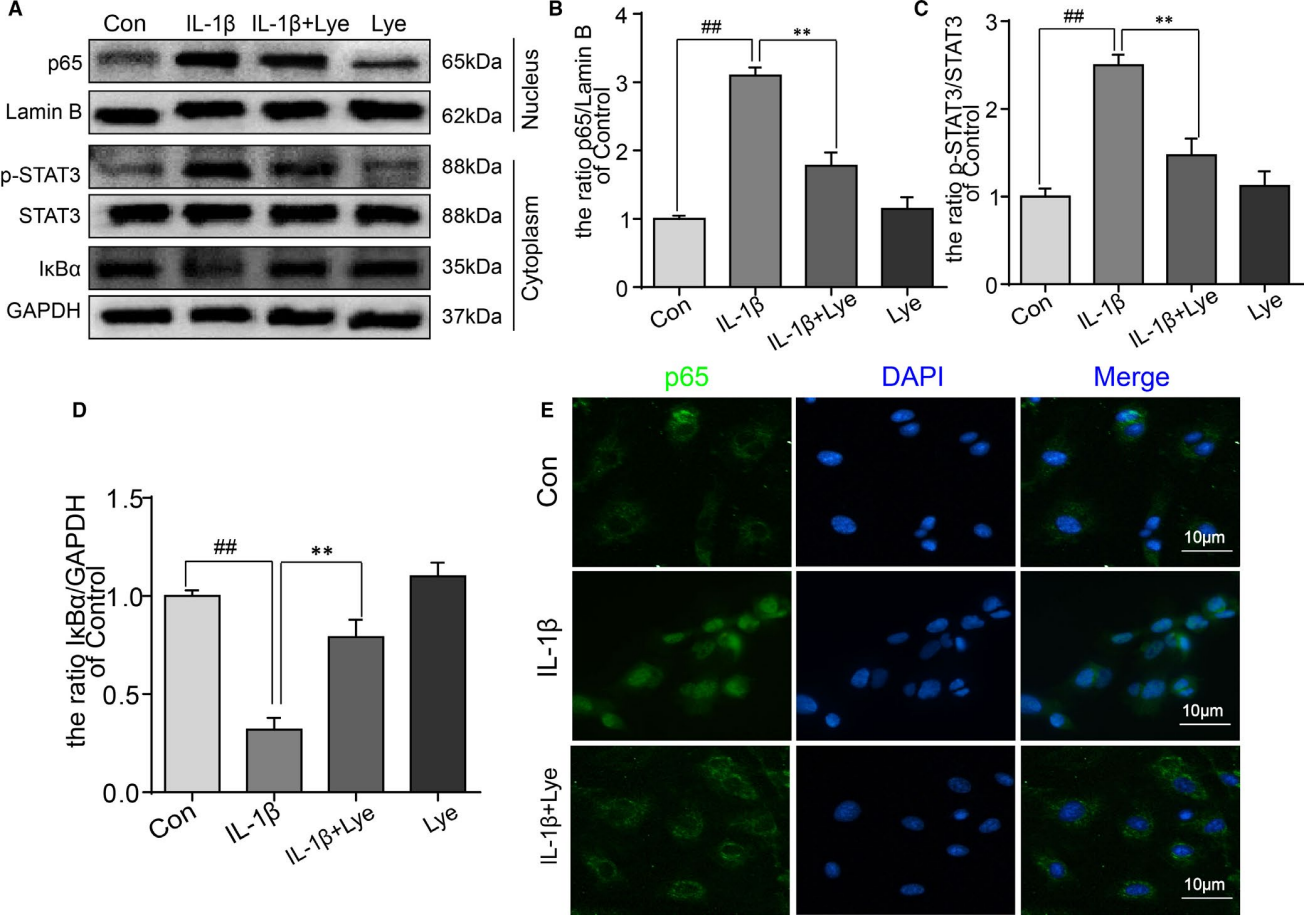
Lye repressed activation of NF‐κB and STAT3 pathway which induced by IL‐1β in chondrocytes. A‐D, The protein expressions of p65, p‐STAT3 and IκBα in chondrocytes with different treatment were analysed by Western blot, and quantified. E, The translocation of p65 from cytoplasm to nucleus was analysed by the immunofluorescence and the nucleus was stained by DAPI (scale bar: 10 μm). All data represent Mean values ± SD ##*P* < 0.01, compared with control group; **P < 0.01, compared with IL‐1β treatment group, n = 5

Further, the NF‐κB (p65) and STAT3 pathways were investigated to explore the mechanism of Lye in anti‐inflammatory setting. With the stimulation of IL‐1β, the level of p65 was evidently increased and this change could be reversed with the Lye pretreatment. At the same time, the trend of IκBα variation were contrary to that of p65 (Figure 4A,B,D). The stimulation of IL‐1β resulted in significant activation of STAT3 phosphorylation; however, the phosphorylation also could be inhibited by Lye (Figure 4A,C). There were no significant differences in the expressions of p65, IκBα and p‐STAT3, when chondrocytes were treated with Lye alone as compared to the control group (Figure 4A‐D). As shown in Figure S1a,b,e, the expression level of p65 and IκBα in both MG132 group and Lye group was significantly different from that in IL‐1β group, but there was no significant difference between MG132 group and Lye group. The result of immunofluorescence showed that the nuclear translocation of p65 after the stimulation with IL‐1β in chondrocytes more evident. As shown in Figure 4E, the location of p65 was almost invisible in the nucleus when there was no IL‐1β. However, when IL‐1β acts on the chondrocytes, the p65 almost expressed in the nucleus. The translocation of p65 from the cytoplasm to nucleus could be suppressed after the pretreatment of Lye. The result of p65 immunofluorescence assay was not contradicting to that of the Western blot, indicating that Lye could inhibit IL‐1β‐induced p65 nuclear translocation. Therefore, we further inferred that Lye repressed the activation of NF‐κB and STAT3 pathway which induced by IL‐1β in chondrocytes to play an anti‐inflammatory role.

### 3.5 Lye suppressed the inflammation and the degradation of ECM in chondrocytes via regulating the Nrf2/HO‐1 signal pathway

**FIGURE 5:**
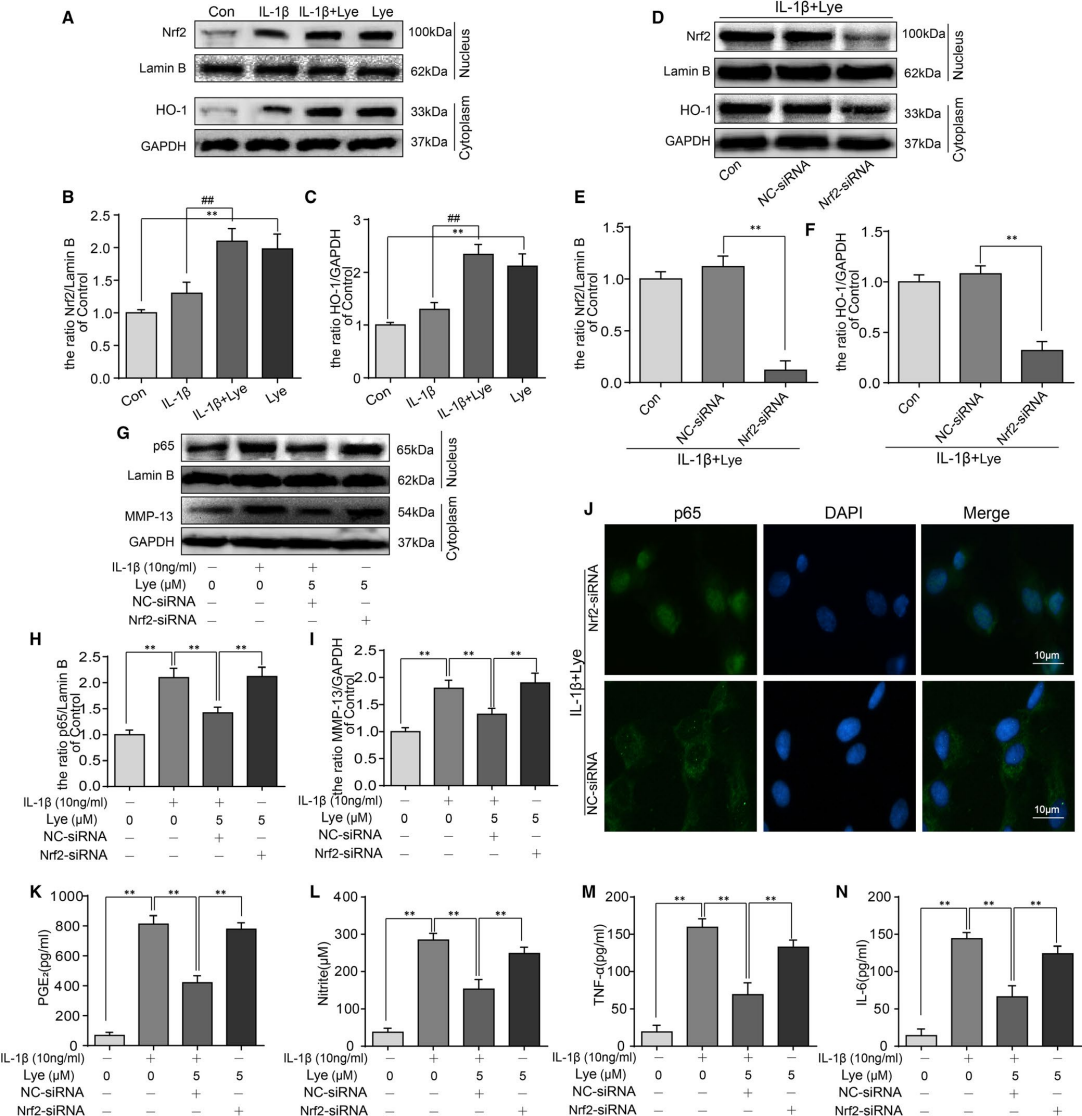
Lye could suppress the inflammation and the degradation of ECM in chondrocytes via regulating the Nrf2/HO‐1 signal pathway. A‐C, The expressions of Nrf2 and HO‐1 in chondrocytes were analysed by Western blot and quantified. D‐I, When Nrf2‐siRNA transfect to chondrocytes, the protein levels of Nrf2, HO‐1, p65 and MMP‐13 were determined by Western blot and quantified. J, The nucleus translocation of p65 was observed by the immunofluorescence staining after transfection of Nrf2‐siRNA (scale bar: 10 μm). K‐N, The changes of PGE2, NO, TNF‐α and IL‐6 in each group were observed by ELISA. Data were represented as mean ± SD. **P < 0.01, n = 5

Treatment with Lye showed an increase in the levels of Nrf2 and HO‐1 on Western bolt regardless of whether or not IL‐1β stimulates the chondrocytes. Contrarily, IL‐1β acting on chondrocytes alone did not cause any evident changes in the expressions of Nrf2 and HO‐1 (Figure 5A‐C). To further comprehend the relationship between the anti‐inflammatory effect of Lye and the Nrf2/HO‐1 signal pathway, before the treatment of Lye, the Nrf2‐siRNA and NC‐siRNA were used to transfect the chondrocytes. As shown in Figure 5D‐F, the Nrf2‐siRNA could significantly inhibit the production of Nrf2 and thus reduce the expression of HO‐1. That is to say that the Nrf2‐siRNA could suppress the Nrf2/HO‐1 signal pathway. The effect of Lye on inhibiting p65 elevation could be reversed by the Nrf2‐siRNA, which was also applied to MMP‐13 (Figure 5G‐I). The result of immunofluorescence assay showed that the Nrf2‐siRNA could disable the effect of Lye on inhibiting p65 nuclear translocation (Figure 5J). The ELISA was also performed after chondrocytes were transfected with the Nrf2‐siRNA and the result was as shown in Figure 5K‐M. The Nrf2‐siRNA could disable the effect of Lye on inhibiting NO, PGE_2_, IL‐6 and TNF‐α expression. In summary, Lye could inhibit IL‐1β‐induced inflammation and activate Nrf2/HO‐1 signal pathway, but the anti‐inflammatory effect could be significantly weakened. However, the Nrf2‐siRNA could inhibit the Nrf2/HO‐1 pathway. Therefore, it could be speculated that Lye played a role in inhibiting IL‐1 β‐induced inflammatory response and degradation of ECM in chondrocytes by activating the Nrf2/HO‐1 pathway.

### 3.6 Lye mitigates the development of OA and enhance the expression of Nrf2 in the mice model of DMM in vivo

**FIGURE 6:**
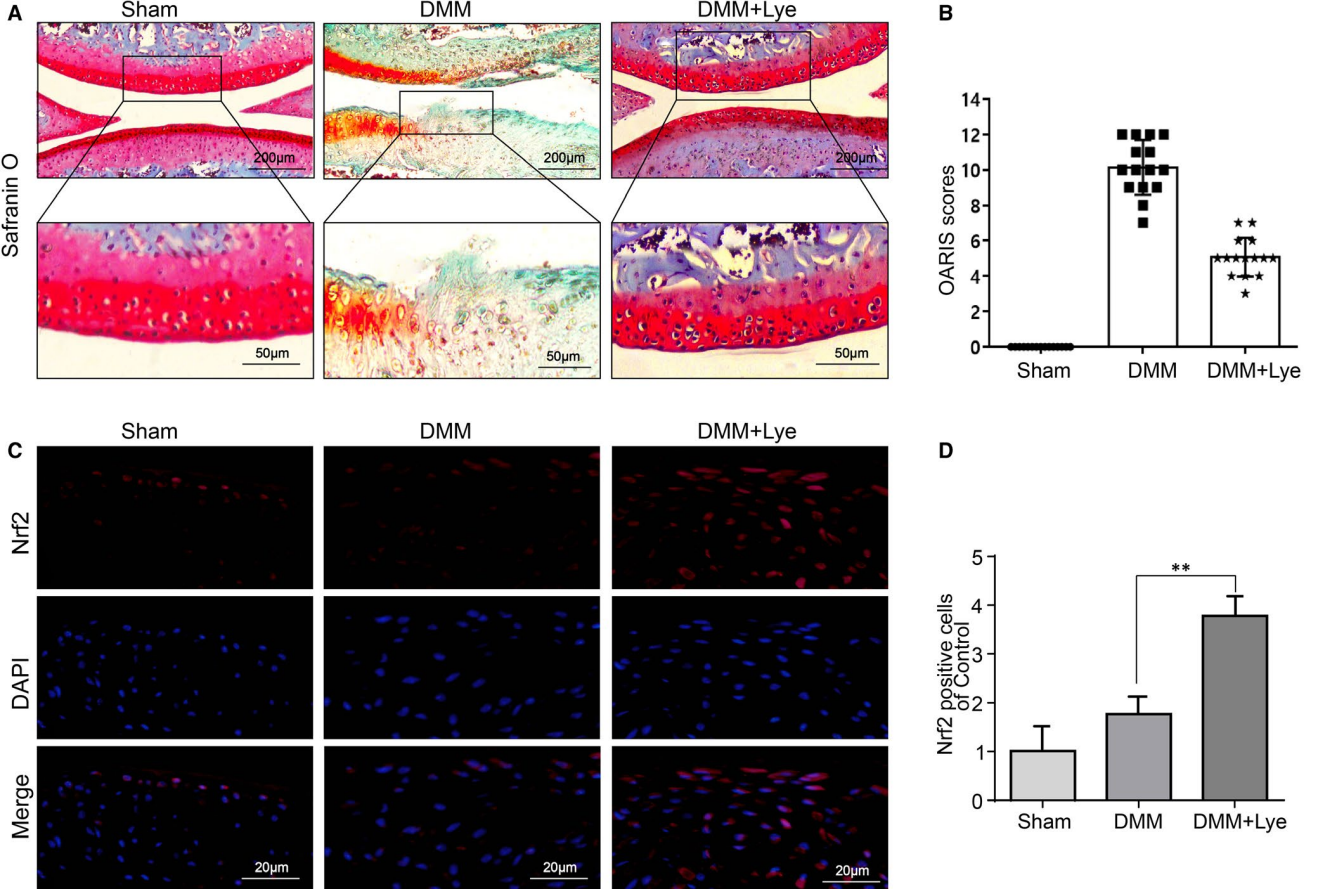
Lye mitigates the development of osteoarthritis and enhances the expression of Nrf2 in the mice model of DMM in vivo. A, The knee joint S‐O staining of each mouse group at 8 weeks after surgery (scale bar: 200 μm). B, The OARIS scores of each group were presented. C, The expression of Nrf2 in the cartilage samples were observed by immunofluorescence staining (scale bar: 20 μm). D, The comparison of Nrf2‐positive cells in total chondrocytes of each group. The data were represented in the figures as mean ± SD **P < 0.01, n = 15

The mice model of DMM were established to evaluate the effect of Lye on OA development in vivo. After the operation, the mice were intragastric administered with Lye (5 mg/kg) daily for 8 weeks. Finally, the knee tissues were stained with Safranin O and evaluated under microscope and scored by OARSI system. As presented in Figure 6A,B, compared with the control group, the erosion of articular surface of the DMM group was evidently more severe. Additionally, the content of proteoglycan was significantly lower than that of the control group. However, it was noted that the flatness of the surface lamina and the content of proteoglycan in the Lye group were significantly better than that in the DMM group, indicating that Lye could suppress the OA development. The scores of OARSI system further illustrated the results of Safranin O staining in a semi‐quantitative manner. It should be noted that there was no significant difference in the flatness of the surface lamina or the content of proteoglycan between the ibuprofen group and the Lye group (Figure S2). Furthermore, the immunofluorescence staining of the knee histologic section for Nrf2 was performed (Figure 6C,D). The luminance of the Nrf2 immunofluorescence was dim in the control group and DMM group; in stark contrast, the number of positive cells was increased significantly because of the treatment of Lye.

## 4 DISCUSSION

The occurrence and development of OA could be regarded as an unbalanced state between chondrocytes, ECM and cytokines to some extent. When tissue injury occurs, the chondrocytes mediate the expression of pro‐inflammatory genes, and overexpression of cytokines such as IL‐1β, TNF‐α, COX‐2 and iNOS.^31^, ^32^, ^33^ These cytokines further activate the downstream inflammatory factors (such as PGE_2_ and NO) and increase the matrix degradation proteases.^34^, ^35^, ^36^ Therefore, the ECM gets destroyed which results in the formation of positive feedback to accelerate joint destruction. At present, the main therapeutic drugs for OA are NSAIDs, although they can only relieve the disease symptoms.^37^

Lye, a type of carotenoid, is currently a popular food ingredient. Many articles have reported that Lye can inhibit inflammation and prevent spermatogenic disorders and nephrotoxicity.^17^, ^22^, ^23^, ^24^, ^25^, ^26^ Additionally, in the daily diet, a large part of Lye comes from tomatoes, watermelons, pink grapefruit and other red vegetables or fruits.^38^, ^39^ Meanwhile, compared with fresh tomatoes, it has been shown that processing the food and cooking the tomatoes could improve the bioavailability of Lye.^40^, ^41^, ^42^ Lye was administered by intragastric treatment in this study. The dose of Lye for mice was 5 mg/kg/d, according to bodyweight and body surface area,^43^, ^44^ which was approximately equal to 0.44 mg/kg/d for a human. Therefore, for a 60 kg adult, the dose of Lye in our animal experiment was approximately equivalent to intake of 300 g of tomato juice.^13^ Our experiments demonstrated that Lye could attenuate IL‐1β‐induced chondrocyte inflammation and ECM degradation by activating Nrf2 pathway and inhibiting the NF‐κB and STAT3 pathway. Meanwhile, the mice experiments proved that Lye could restrict the development of OA.

**FIGURE 7:**
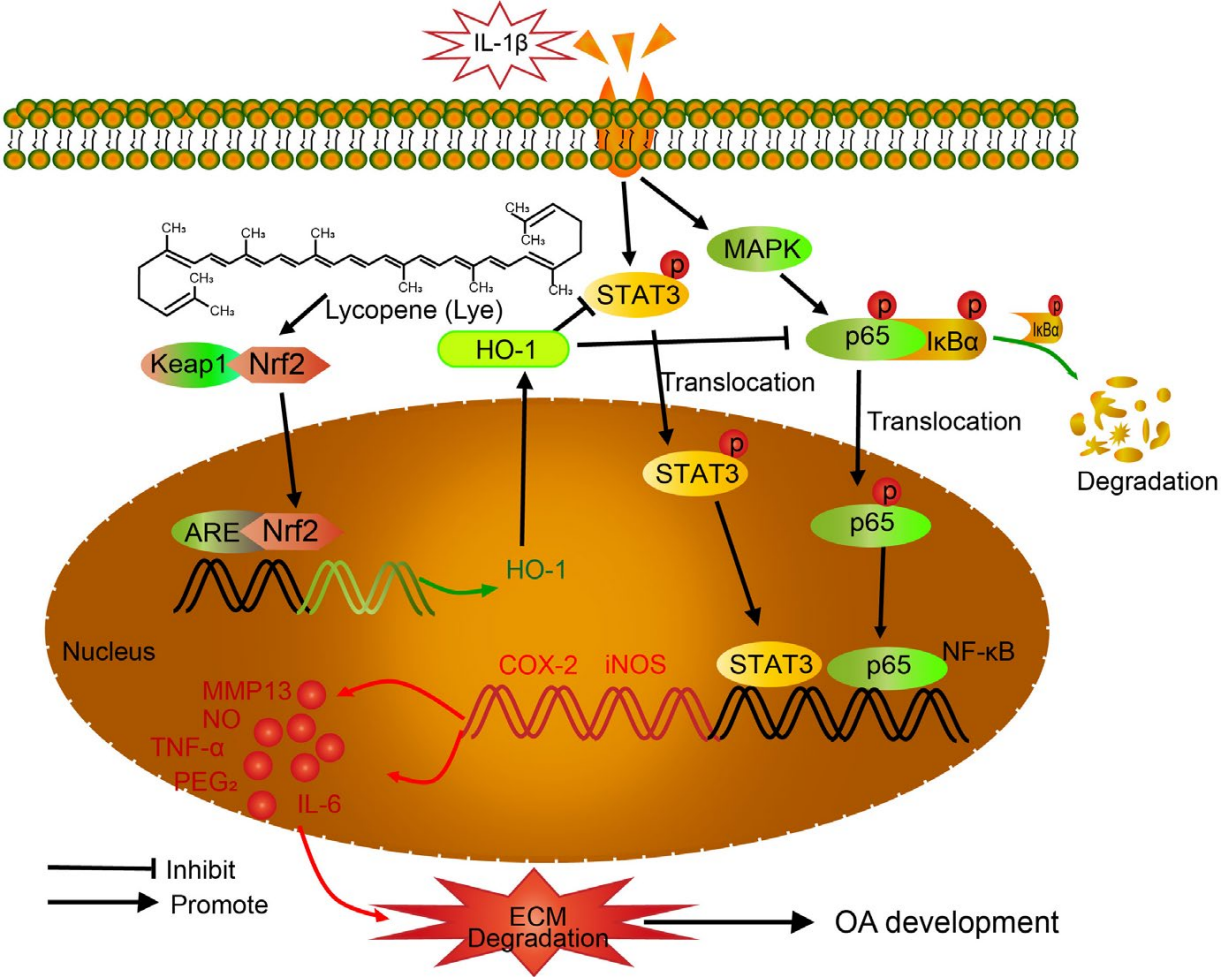
Diagram of the potential protective role of Lye in progress of osteoarthritis

In the studies of the mechanism of OA, NF‐κB axis was a classical signalling pathway that plays a crucial role. As show in Figure 7, IL‐1β acts as a stimulus and induces phosphorylated IκBα‐p65 binding. The p65 then translocate from cytoplasm to nucleus, activating the related genes to express TNF‐α, IL‐6, iNOS, COX‐2 and other products.^44^, ^45^ The expression of PGE_2_ and NO could be induced by COX‐2 and iNOS, respectively,^46^ which would not only further promote the synthesis of ECM degradation markers such as MMPs and ADAMTS5,^32^, ^47^ and ECM catabolism but also inhibit the synthesis of aggrecan and collagen II.^11^ In this study, we found that Lye could down‐regulate the expression of COX‐2 and iNOS at the gene and protein level, inhibit the excessive production of PGE_2_ and NO, and reduce the secretion of TNF‐α and IL‐6. Additionally, we also observed that the regulations of these pro‐inflammatory mediators were related to inhibition of Lye in NF‐κB signal pathway. This was consistent with previous study^48^ that Lye could inhibit the production of TNF‐α and IL‐6 by blocking NF‐κB in bovine mammary epithelial cells.

In addition, STAT3 pathway was abnormally activated in OA,^49^ which played a key role in the MMP13 expression in the chondrocytes.^50^ Previous studies have shown that Lye could inhibit the expression of inflammatory mediators by affecting the STAT3 pathway.^51^, ^52^ Consistent with previous results, our study demonstrated that IL‐1β could induce STAT3 phosphorylation, while Lye could reverse the activation of STAT3 pathway and down‐regulate the expression of MMP‐13. However, Lye alone could not significantly decrease the expression of p‐STAT3, indicating that Lye did not inhibit the normal expression of STAT3 pathway, but reversed its overexpression (Figure 7).

Previous studies have shown that Lye can inhibit the expression of NF‐κB signal pathway by activating Nrf2 pathway.^26^, ^53^ It was also shown that the stimulation of di‐(2‐ethylhexyl)‐phthalate (DEHP) could increase the expression of reactive oxygen species (ROS) in Leydig cells and then activates the Nrf2 signal system, whereas the expression of ROS and Nrf2 would be decreased after the application of Lye.^25^ In our experiment, we observed that Lye could increase the expression of Nrf2 and HO‐1, and decrease the nuclear expression of p65, suggesting that Lye could activate the Nrf2/HO‐1 system and significantly inhibit the nuclear translocation of p65 (Figures 4, 5 and 7). In order to further determine the interaction of Lye, Nrf2 pathway and p65 nuclear translocation, Nrf2‐siRNA was transfected into chondrocytes to inhibit Nrf2 pathway. Finally, the results showed that while the Nrf2 pathway was inhibited, the effect of Lye on inhibiting p65 nuclear translocation was significantly weakened, suggesting that Lye mediated inflammatory response and ECM degradation by activating Nrf2 pathway. The effect of Lye on Nrf2 signal system observed in this study was contradictory to the results of study by Zhao et al,^25^ who evaluated the antioxidant effect of Lye. In their study, DEHP was used as an indirect stimulus to increase the level of ROS in Leydig cells, and ROS was considered as a new stimulator to activate the related pathway. Thus, the body up‐regulated the expression of Nrf2 in order to eliminate the ROS. When Lye was added, the production of ROS induced by DEHP could be inhibited before the activation of Nrf2, which activated was by ROS; thus, the expression of Nrf2 was weakened. The focus of our study was on the anti‐inflammatory effect of Lye. The IL‐1β was a direct stimulating factor in our experiment, which induced the production of inflammatory factors via a series of pathways and did not affect the expression level of Nrf2 significantly (Figure 5A). Lye played a role in this process by activating the Nrf2 pathway to inhibit NF‐κB system. Additionally, in the experiment by Zhao, et al, the level of Nrf2 in Leydig cells did not change significantly after treatment with Lye alone. In our experiment, the Nrf2 level of chondrocytes treated with Lye alone were significantly higher than that of the control group. This may be as a result of the different cells selected in the two experiments. In summary, the difference between the two experiments may be as a result of the different mechanisms involved in anti‐oxidation and anti‐inflammation of Lye or the different types of cells used.

HO‐1, the downstream protein of Nrf2, was the rate‐limiting enzyme of haem degradation and it promoted the decomposition of haem into CO, ferrous ion and biliverdin. These end products had antioxidant and anti‐inflammatory effects.^54^, ^55^, ^56^ Previous literatures have reported that the interaction of Nrf2/HO‐1 and NF‐κB pathways jointly regulates the inflammatory process.^57^ In this experiment, it was found that Lye increased the expression of HO‐1 in cytoplasm by activating Nrf2, thus inhibiting NF‐κB signal pathway and STAT3 system, and thereby achieving an anti‐inflammatory effect (Figure 7).

At the histological level, the OA showed narrowing of articular space, uneven articular surface, erosion of articular cartilage, etc.^3^ We found that these manifestations could be reversed by Lye, suggesting that Lye could relieve the pain and disease development in OA patients to some extent. The OARSI system,^30^ which is widely used as a scoring system to evaluate the severity of OA, was used to analyse the results of animal experiment in a semi‐quantitative way. These conclusions were consistent with cell experiments, indicating that Lye could inhibit the progression of OA in vivo.

Overall, our study found that Nrf2 pathway could be activated by Lye to antagonize the inflammatory response and the degradation of ECM in chondrocytes, which was regulated by NF‐κB and STAT3 pathway. We also observed that Lye could ameliorate the OA development of DMM mouse model after intragastric administration.

## 5 CONCLUSION

In brief, this study provides a new direction for the treatment of OA. The change in the daily dietary habits to include more Lye‐rich fruits and vegetables or Lye‐based products could benefit in condition such as OA.

## 6 ETHICAL APPROVAL AND CONSENT TO PARTICIPATE

This study was approved by Wenzhou Medical University Animal Care and Use Committee (ethic code: 2018‐078).

## CONFLICTS OF INTERESTS

The authors confirm that there are no conflicts of interest.

## AUTHOR CONTRIBUTION

**Jingdi Zhan:** Writing‐original draft (equal); Writing‐review & editing (equal). **Zijian Yan:** Data curation (equal). **Xiaojiang Kong:** Conceptualization (equal); Methodology (equal). **Junling Liu:** Investigation (equal); Visualization (equal). **Zeng Lin:** Conceptualization (equal); Methodology (equal). **Weihui Qi:** Software (equal); Validation (equal). **Yifan Wu:** Data curation (equal); Software (equal). **Jian Lin:** Software (equal); Validation (equal). **Xiaoyun Pan:** Supervision (equal); Writing‐review & editing (equal). **xinghe Xue:** Investigation (equal); Supervision (equal); Visualization (lead).

## Supporting information

Figure S1

Figure S2

Supplementary Material

## Data Availability

The data that support the findings of this study are available from the corresponding author upon reasonable request.
