# Supplementary figures and images for: Lycopene inhibits IL‐1β‐induced inflammation in mouse chondrocytes and mediates murine osteoarthritis
Source: J Cell Mol Med. 2021 Mar 10;25(7):3573–84. doi: 10.1111/jcmm.16443 (PMC8034440; doi:10.1111/jcmm.16443)

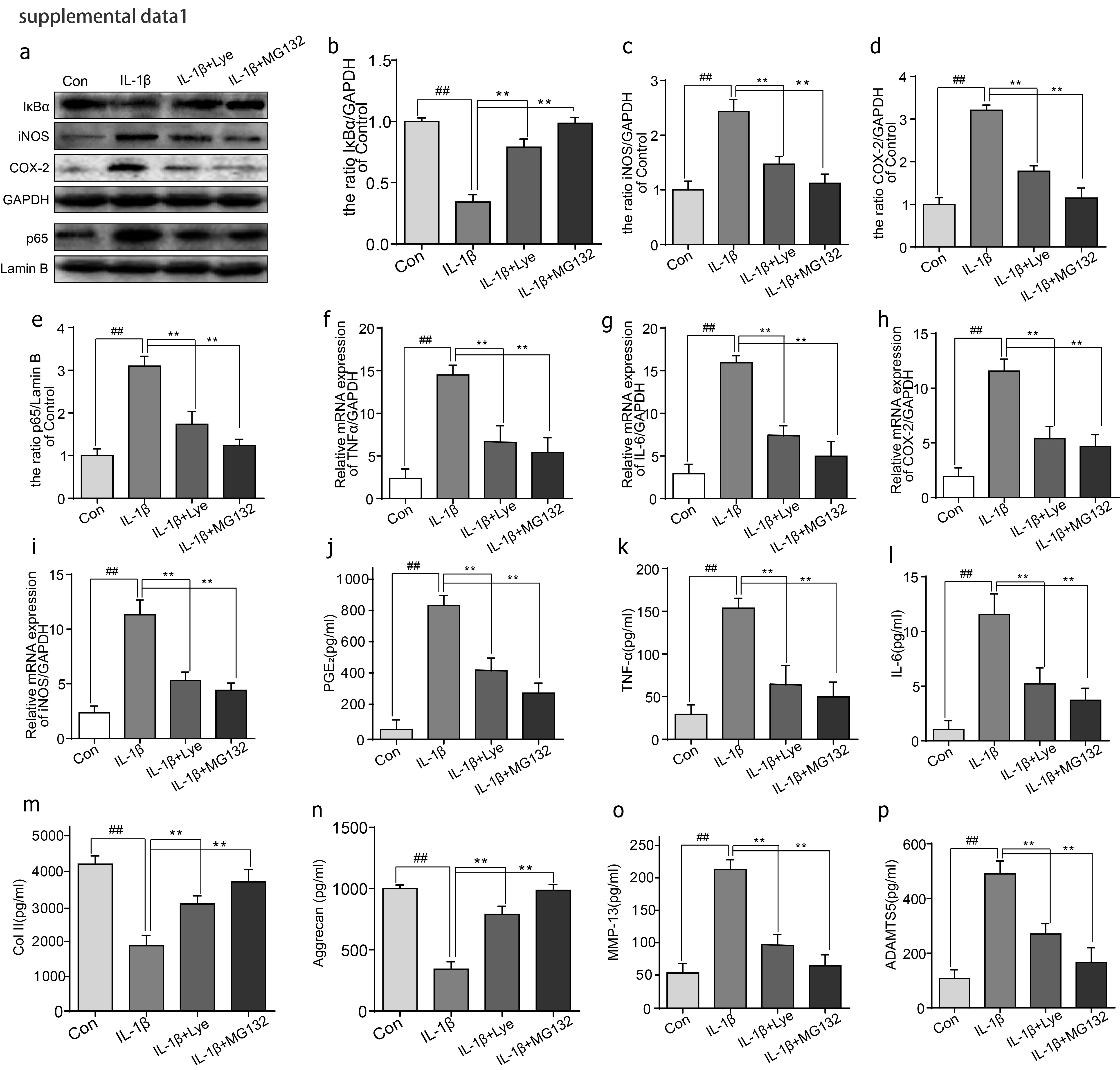

Supplement: Supplementary file 1 — Figure S1 [file JCMM-25-3573-s002.tif]

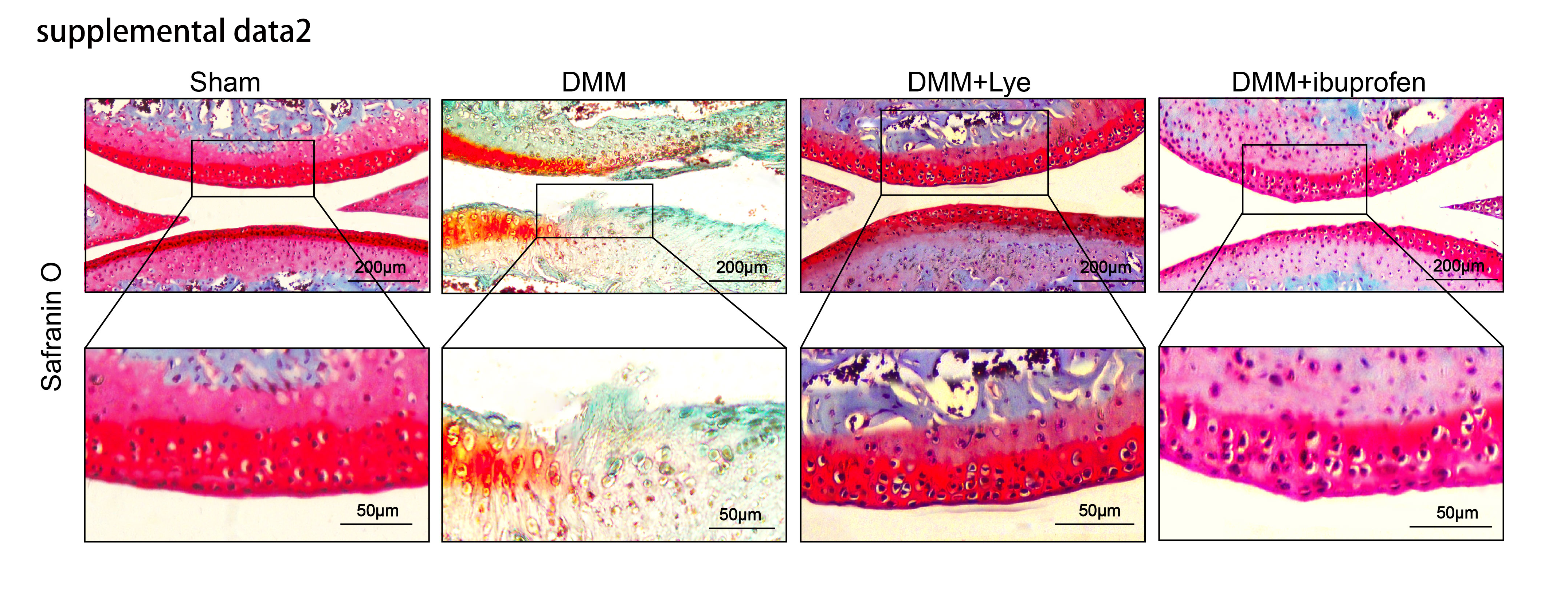

Supplement: Supplementary file 2 — Figure S2 [file JCMM-25-3573-s003.tif]
