# Supplementary material for: Lycopene inhibits IL‐1β‐induced inflammation in mouse chondrocytes and mediates murine osteoarthritis
Source: J Cell Mol Med. 2021 Mar 10;25(7):3573–84. doi: 10.1111/jcmm.16443 (PMC8034440; doi:10.1111/jcmm.16443)
Supplement: Supplementary file 3 — Supplementary Material [file JCMM-25-3573-s001.docx]

Figure S1: Comparison of the inhibitory effect of Lye and MG132 on chondrocyte inflammation. (a-e). The expressions of IκBα iNOS, COX-2 and p65 at protein level were detected by western blot with IL-1β, Lye and MG132. (f-i). The expression of mRNA of TNF-α, IL-6, COX-2 and iNOS were measured via real-time PCR. (j-p). Effects of Lye and MG132 on production of IL-1β-induced PGE2, NO, TNF-α and IL-6 were detected by ELISA. All data represent Mean values ± S.D. ##P< 0.01, compared with control group; **P< 0.01, compared with IL-1β treatment group, n = 5.

Figure S2: The knee joint S-O staining of each mouse group. There is no significant difference in knee surface between the Lye group and the ibuprofen group.
